# Quantitative 3-D head ultrasound measurements of ventricle volume to determine thresholds for preterm neonates requiring interventional therapies following posthemorrhagic ventricle dilatation

**DOI:** 10.1117/1.JMI.5.2.026001

**Published:** 2018-06-26

**Authors:** Jessica Kishimoto, Aaron Fenster, David S. C. Lee, Sandrine de Ribaupierre

**Affiliations:** a University of Western Ontario, Department of Medical Biophysics, London, Ontario, Canada; b University of Western Ontario, Robarts Research Institute, Imaging Research Laboratories, London, Ontario, Canada; c University of Western Ontario, London Health Sciences Centre, Department of Clinical Neurological Sciences, London, Ontario, Canada

**Keywords:** three-dimensional ultrasound, cranial ultrasound, preterm neonate, neurosurgery, hydrocephalus

## Abstract

Dilatation of the cerebral ventricles is a common condition in preterm neonates with intraventricular hemorrhage. This posthemorrhagic ventricle dilatation (PHVD) can lead to lifelong neurological impairment through ischemic injury due to increased intracranial pressure, and without treatment can lead to death. Two-dimensional ultrasound (US) through the fontanelles of the patients is serially acquired to monitor the progression of PHVD. These images are used in conjunction with clinical experience and physical exams to determine when interventional therapies such as needle aspiration of the built up cerebrospinal fluid (ventricle tap, VT) might be indicated for a patient; however, quantitative measurements of the ventricles size are often not performed. We describe the potential utility of the quantitative three-dimensional (3-D) US measurements of ventricle volumes (VVs) in 38 preterm neonates to monitor and manage PHVD. Specifically, we determined 3-D US VV thresholds for patients who received VT in comparison to patients with PHVD who resolve without intervention. In addition, since many patients who have an initial VT will receive subsequent interventions, we determined which PHVD patients will receive additional VT after the initial one has been performed.

## 1 Introduction

Despite advances in neonatal care and improved evidence-based perinatal management guidelines, preterm neonates are still at a high risk of morbidity. Intraventricular hemorrhage (IVH), bleeding inside the cerebral ventricles, has been decreasing in prevalence yet remains as a common morbidity among preterm born neonates.^1^ The risk of long-term disabilities such as cerebral palsy and cognitive impairment increases significantly with the occurrence of posthemorrhagic hydrocephalus (PHH), the abnormal enlargement of the ventricles, which typically occurs in 25% to 28% of IVH patients with severe bleeds (grades III and IV by the Papile scale^2^).^3^ In general, PHH is thought to be caused by the blood clots and inflammatory reactions from blood breakdown products causing obstruction of the flow of CSF, which normally flows from the ventricles, where CSF is generated to the subarachnoid space where it is reabsorbed into the blood stream.

While the definitive treatment for PHH is the placement of a ventriculoperitoneal shunt (tubing between the ventricles and the abdomen), placement of the shunt is often delayed by several weeks to allow for the blood clot to resolve, the CSF proteins to decrease (<1000  mg/dL), and the weight of the infant to increase (at minimum >800  g to 1 kg). However, during this delay in treatment, the increase in ventricle size, also known as posthemorrhagic ventricle dilatation (PHVD), can cause dangerously increased intracranial pressure (ICP), which can limit blood flow to the brain. Any ischemia or decreased blood flow during this critical period can be detrimental to the developing brain and can lead to impairment later in life. Due to this risk, neonates who have rapidly progressing PHVD and some with persistent, slowly progressing PHVD will require temporary interventions to relieve the elevated ICP. These interventions include lumbar/ventricle puncture, external ventricle drainage, ventricular access devices (reservoirs), and ventriculosubgaleal shunts. There is no consensus of evidence as to which of these methods are the best for temporary CSF diversion.^4^^,^^5^

Currently, clinical experience as well as daily head circumference and transfontanelle brain ultrasound (US) are used to determine when neonates receive temporary interventions to removed built up CSF.^6^ However, it has been shown that head circumference does not accurately correlate with increase in clinical US measurements (Evan’s ratio)^7^ or with ventricular volume.^8^ In addition, clinical signs of increased ICP (apnea and bradycardia) are nonspecific in the preterm infant and could be due to other comorbidities. Early detection of interventional necessity could potentially lead to better management of the condition, while early detection of resolution could provide comfort to the clinician and parents that intervention is unlikely to be necessary. In addition, since many cases of PHVD resolve after a temporizing CSF diversion procedure and do not then require a shunt, we aimed to characterize an US-based metric to determine the resolution of PHVD versus continual, progressive ventricle dilatation leading to PHH, which may receive additional interventions.

Previous work with transfontanelle brain three-dimensional (3-D) US has been attempted in neonates.^9^^–^^13^ While this imaging technique was shown to be feasible and gives similar measurements^14^ and diagnosis^12^^,^^15^^,^^16^ as two-dimensional (2-D) US, its clinical utility beyond 2-D has not been established. Recent studies have had success acquiring 3-D US images of neonatal ventricles using commercial systems;^9^^,^^10^^,^^12^^,^^14^^–^^16^ however, these transducers are not part of standard-of-care and must be purchased separately. The expense of these systems may limit their use, especially in the developing world. A system that could be used in conjunction with a conventional clinical 2-D transducer to generate full 3-D images would allow centers without a 3-D US capable machine to confidently acquire 3-D ventricle volumes (VVs). As hydrocephalus is far from only an issue in the developed world with 6000 cases per year diagnosed in East Africa,^17^ we developed a relatively low-cost 3-D US system that can be modified to generate 3-D US images from virtually any conventional 2-D US system.^18^ From these 3-D images, we measure the volume of the infants’ ventricles. This system has been validated both using test phantom ventricles of known volume^18^ and *in vivo* against the volume of CSF withdrawn during a tap as well as against MRI.^19^

In this paper, we present for the first time a preliminary study used to examine whether a 3-D US-based VV and VV change measurements allow us to prognosticate, which infants with PHVD will receive a temporary intervention and who will have a spontaneous resolution of PHVD.

## 2 Materials and Methods

As part of a larger study, between April 2012 and May 2016, after an initial diagnosis of IVH^1^ was made during standard screening head US exam, preterm neonatal patients were enrolled with informed parental consent. 3-D US images and all experimental protocol were acquired in accordance with a protocol approved by the Research Ethics Board at the University of Western Ontario. Imaging was performed twice per week with 3 to 4 days between imaging sessions.

Interventions were performed based on clinical judgement, with the clinician using the head circumference growth over time, the increase in ventricular dilatation on the 2-D US, as well as sometimes, but not necessarily, clinical signs and symptoms of increased ICP, such as apnea, bradycardia, and a full, tense fontanelle. Interventions were not based on 3-D US images collected during the study, as the clinical team was blinded to all 3-D US images during the course of the stay in the neonatal intensive care unit (NICU). The initial intervention at our center is a ventricular tap (VT) since it can be done at the bedside and does not require an operating room (whereas the Ommaya catheter insertion does); but if taps are thought to be needed frequently, then an external ventricle drain is usually inserted. For the purpose of this study, only patients born <32-weeks gestational age (GA) were included in the analysis.

### 2.1 Three-Dimensional US Image Acquisition

Images were acquired using a Philips HDI 5000 US machine with a C8-5 transducer. 3-D US imaging was performed after placing the US transducer in a handheld motorized housing that tilted the transducer about the axis at the probe tip for 8 to 9 s.^20^ This approach avoids the use of free-hand scanning using any external tracking such as electromagnetic or optical, as these methods require additional more expensive technology and are subject to geometric artifacts due to environmental constrains and line of sight. Furthermore, 3-D scanning without tracking or a motorized fixture is not recommended for accurate volume measurements from 3-D US images.^21^

During image acquisition, 2-D US images were collected on a laptop computer as the motor tilted the transducer over 9.6 s and in-house software reconstructed the 3-D US image in real-time as images were received into the computer. Technical details can be found in Ref. 18. This system has been validated both for geometric validity and volume measurements.

### 2.2 Three-Dimensional US Image Segmentation

**Fig. 1:**
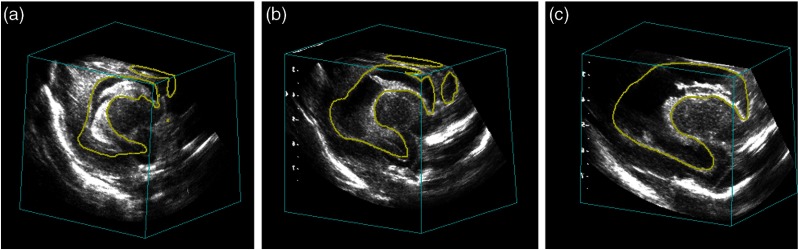
3-D US images of a preterm neonate with PHVD over the first 3 weeks of life who required multiple VT to treat the rapid increase in ventricle size. (a) Week 1, (b) week 2, and (c) week 3. Ventricles have been segmented as indicated by the yellow outline.

The lateral ventricles were manually segmented in parallel sagittal slices with 1 mm gaps between adjacent slices. A trained observer (JK) segmented all the images and a pediatric neurosurgeon verified the boundaries (SdR). Each image required between 20 and 45 min to segment. The 3-D US imaging system and ventricle segmentation have been previously validated using phantom and patient images.^18^ An example of segmented ventricles from a patient with PHVD following IVH is shown in Fig. 1.

### 2.3 Determining Which Neonates with PHVD Received an Initial Ventricle Tap

We performed the analysis of patients who did and did not receive interventional therapies for PHVD. The VV for each scan was recorded during the first month of life for each patient as well as whether or not they eventually received a VT.

#### 2.3.1 Volume measurement as predictive marker

The VV for age ranges <7, 8–11, 12–15, 16–19, 20–22, and 23–27 days of life was calculated and recorded for each patient. Volume measurements were not always attainable for every patient at each time point, as some enrolled late, or were too unstable to be scanned that day. Given the study population leaned toward extremely preterm neonates with at minimum an IVH and often with other morbidities related to prematurity, instability following minimal handling (such as during a head US) was more common in this population than it would be for most NICU patients. For some patients, two measurements were made within the time periods (i.e., days 8 and 11) and both were used as independent points.

#### 2.3.2 Maximum rate of change of ventricle volume

We calculated the rate of change of VV between two scans, and analyzed it for the first week (day <8), second week (days 8 to 14), and third week (days 15 to 21) of life. The neonatal “week of life” was categorized depending on the week of the second scan (i.e., if scans were obtained on days 3 and 7 of life, this would be considered “first week of life” but if scans were obtained on days 6 and 10 of life, this would be “second week of life”).

### 2.4 Determining Which Neonates with PHVD Will Receive Follow-Up Interventions

Since most patients with PHVD who have an initial VT will go on to have multiple VTs during the course of the stay in the NICU, we investigated whether or not image-based measurements could detect whether further treatments would be received. Thus, we analyzed VV as well as VV change using the images obtained closest to the initial VT but not on the day of, and within the week after the VT to see if we could establish a difference among these patients.

### 2.5 Comparison to 2-D US-Based Thresholds for Intervention

Many centers opt to use 2-D US-based measurements as thresholds for intervention. Such measurements have been described by Brouwer et al.,^22^and we will be using their cross-sectional reference curves in this study as they include neonates as young as 25-weeks GA. The “action” threshold we will report on is the ventricle index (VI) 97th centile +4  mm.^23^ As we did not use any 2-D US measurements to determine interventions during the study period, we were perhaps far more conservative in intervening in these patients than most clinicians would have been. Thus, we can retrospectively investigate at what point the neonates in our study would have crossed 2-D US image-based thresholds in comparison to the 3-D US-based thresholds. Thus, we report the day of life a patient crossed over an image-based threshold. As 2-D US VI thresholds were generated using an independent data set, we directly used our measurements against the indicated threshold to report the first day of life the patient had 2-D US-based measurements above the VI 97th +4  mm if it occurred, otherwise, the tested date would be reported.

We used leave-one-out cross-validation to investigate 3-D US-based thresholds. Patient images were binned into time points previously alluded to (<7, 8 to 11, 12 to 15, 16 to 19, 20 to 22, and 23 to 27 days of life). Some patients had multiple images within the bin, and if this patient was “left out” all their images were removed from the test set, and a new threshold was generated to be tested against and the test patient that was reported to be above (interventional) or below (noninterventional) the threshold. Test age of life varied based on when patient was enrolled in the study, with the earliest scan date used as the test date. This process was repeated separately for all patients.

### 2.6 Data Analysis

Receiver operator curves (ROC) were generated among patients who did and did not receive interventions using 3-D US-based VVs. In the second study in patients who had previous interventions, ROC analysis was performed using 3-D US-based VVs, among patients who did not receive further interventions, and those that received at least one additional intervention. For each parameter (volume and volume change), optimal threshold for intervention was estimated using the highest product of sensitivity and specificity. In addition, the area under the ROC curve (AUC), sensitivity, and specificity were calculated. Leave-one-out thresholds were generated using the same method. Assuming a Bonferroni correction, two-sided t-tests were performed between all previously described compared groups of patients with an α=0.05. All analyses were performed using GraphPad Prism 6 (GraphPad Software, San Diego, California).

## 3 Results

### 3.1 Patient Characteristics

Thirty-eight neonates were enrolled prior to the second week of life (before most patients with PHVD would receive an intervention) and were used to determine thresholds in this study. Of those, 14 patients received at least one VT. Mean age of enrolment was 9 days of life, and 12 patients were enrolled in the first week of life (seven did not receive interventions, five had at least a single VT). Descriptions of the all study patients are shown in Table 1.

**Table 1:** Patient characteristics for those who did and did not receive interventional therapies.

	Intervention (N=14)	No intervention (N=24)
Gestational age (weeks±SD)	27.2±2.3	27.3±2.7
Birth weight (g±SD)	1060±300	1040±490
Sex (M/F)	10/4	16/8
IVH grade^3^ (I/II/III/IV)	0/1/7/6	3/13/6/2

### 3.2 Determining Which Neonates with PHVD Receive an Initial Ventricle Tap

#### 3.2.1 Volume measurement as a predictive marker

**Fig. 2:**
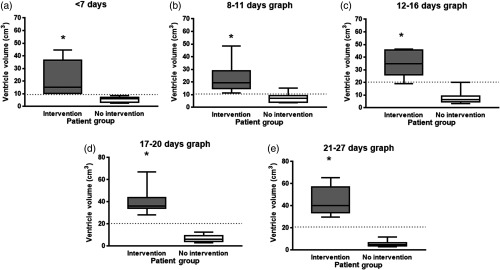
Box and whisker plots of the VV of neonatal patients who received an interventional ventricle tap for clinical reasons (intervention), compared to those who did not receive intervention (no intervention). Images obtained at (a) 7, (b) 8 to 11, (c) 12 to 16, (d) 17 to 20, and (e) 21 to 27 days of life for patients born <32-weeks GA. Threshold from ROC analysis is marked as a dotted line. Significant differences from t-test are indicated as * over the graph.

Table 2 shows the threshold in VV used to determine which patients did or did not receive a VT intervention for PHVD for a given patient’s age in days. Sensitivity and specificity for the threshold used as well as AUC are also given in Table 2. Figure 2 shows the VV in neonates with PHVD during the different time periods previously specified as well as the calculated threshold from ROC analysis. In general, using VV, we found the thresholds that characterized patients who received interventional therapy with high sensitivity (100% to 91.7%) and high specificity (100% to 92.9%) during the first three weeks of life (Table 2). The most ambiguity came during the second week of life (Table 2, 8 to 11 and 12 to 16 days) when a portion of the patients who did not receive interventions had transient increases in ventricle size, reflected in a small increase in mean volume from 7 to $8\;{\mathrm{cm}}^{3}$. After this time, for those patients who did not receive interventions but had transient, small increases in VV had VVs that eventually decreased or stabilized to on average be about $7\;{\mathrm{cm}}^{3}$.

**Table 2:** Optimal sensitivity and specificity using the maximum single measurement of 3-D US-based VV, AUC, and volume threshold obtained from ROC curve specificity/sensitivity maximum. The number of patients for each time interval is indicated as recruitment often happened after the first week of life, and not all patients were stable enough to image at every time interval.

Age of patient	Interventional patients (N)	No interventions (N)	Sensitivity (%)	Specificity (%)	AUC	Threshold used (${\mathrm{cm}}^{3}$)
<7 days	7	8	100	100	1	>9.4
8 to 11 days	14	12	91.7	100	0.98	>10.6
12 to 16 days	14	22	100	92.9	0.99	>20.3
17 to 20 days	8	13	100	100	1	>20.4
21 to 27 days	5	17	100	100	1	>20.9

The patients who had interventions saw a continued increase in VV at every time interval and this can be seen in Fig. 2. The group differences among patients who did and did not receive interventions were reflected in the increase in the threshold between the two groups, as well as the increase in sensitivity and specificity after day 17 (Table 2).

#### 3.2.2 Maximum rate of change of ventricle volume as a predictive marker

**Fig. 3:**
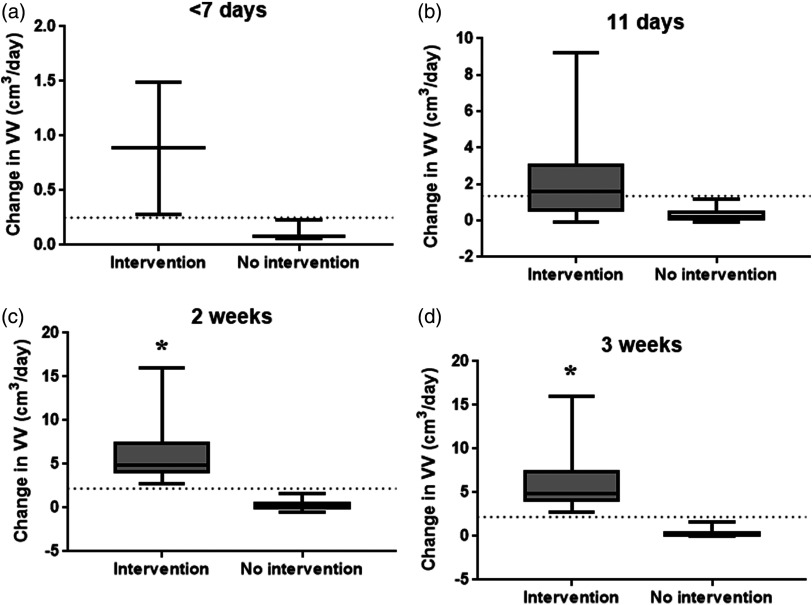
Box and whisker plot of the rate of change in VV between consecutive imaging sessions for preterm neonates born <32-weeks GA who received an interventional ventricle tap for clinical reasons (intervention), compared to those who did not receive intervention (no intervention). Scans taken at (a) 1 week of life, (b) 11 days of life, (c) second week of life, and (d) third week of life. Threshold from ROC analysis is marked as a dotted line. Significant differences from t-test are indicated as * over the graph.

Table 3 shows the threshold in the rate of change VV to determine, which patients did or did not receive a temporary intervention for PHH for different age ranges in days. Sensitivity and specificity for the threshold used as well as the AUC are also given in Table 3. Figure 3 shows the rate of change in VV in neonates with PHVD between those who did and did not receive interventions during the different age ranges previously specified. In addition, the threshold calculated in ROC analysis is shown in Fig. 3 for ease of comparison between time periods.

**Table 3:** Optimal sensitivity and specificity using the maximum rate of change in the first 3 weeks of life in 3-D US VV is reported along with AUC from ROC curve, and the threshold is reported from ROC curve specificity/sensitivity maximum. The number of patients for each time point is indicated as recruitment often happened after the first week of life, or patients only had a single VV recorded during their time in the study.

Age of patient	Interventional patients (N)	No interventions (N)	Sensitivity (%)	Specificity (%)	AUC	Threshold used (${\mathrm{cm}}^{3}/\mathrm{day}$)
<7 days	2	3	100	100	1	>0.25
<11 days	8	9	100	75	0.82	>1.35
2 weeks	10	18	100	100	1	>2.20
3 weeks	10	19	100	100	1	>2.20

The rate of change in VV was highly sensitive for detecting which patients received interventions (100% sensitivity) during all age intervals monitored (Table 3). The ROC in VV additionally allowed for more specific thresholds (100% specificity) to be generated for patients who received interventional therapy in comparison to a single measurement of VV for all age ranges except for those of <11  days, which had a sensitivity of 75% (Table 3). From days 7 to 11, some patients who did not receive intervention had a transient period of ventricle dilatation that appeared to resolve by the second week of life when the rate of change stopped increasing or increased by a small amount (Fig. 3). The threshold for 7 days of life had a very low N (five patients total), which should be taken into consideration as this might not be representative of the total patient group studied (Table 3).

### 3.3 Determining Which Neonates with PHVD Will Receive Follow-Up Interventions

**Fig. 4:**
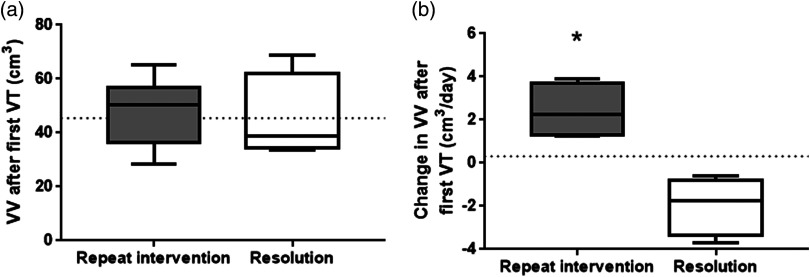
(a) Box and whisker plot of the VV in the imaging session immediately after VT as well as (b) the change in VV in consecutive imaging session after VT for patients who received either multiple interventions, or had resolving ventricle dilatation after a single intervention. Threshold from ROC analysis is marked as a dotted line. Significant differences from t-test are indicated as * over the graph.

For this study, we examined only the patients who already had a previous VT. Of the 11 patients with a previous VT, four resolved clinically without further interventions during their NICU stay. Table 4 shows 3-D US-based thresholds among patients who received at least one additional VT, and patients who had PHVD, which resolved after a single VT. Figure 4(a) shows the 3-D US-based VV as well as the change in VV [Fig. 4(b)] among neonates who previously had VT who received repeated VT and those who had resolving PHVD. Ventricle volume appeared to remain high in 3-D US images acquired after a first intervention, and there was no clear threshold among those patients who received further interventional management [Fig. 4(a)]. Indeed, there was no significant difference between the average VV for patients who received additional VT ($48.8\pm 12.5\;{\mathrm{cm}}^{3}$ SD) and patients who were undergoing resolution of PHVD ($45.1\pm 16.1\;{\mathrm{cm}}^{3}$ SD). However, when the patient was monitored for an additional imaging session and the change of volume could be calculated, it was clear that patients who did not receive further VT had a decrease in ventricle size [Fig. 4(b)]. The threshold for change in VV ($<0.31\;{\mathrm{cm}}^{3}/\text{day}$) was able to characterize patients who were resolving versus those receiving further monitoring with a sensitivity and specificity of 100% (Table 4).

**Table 4:** Optimal sensitivity and specificity using the maximum VV and maximum rate of change in VV (ΔVV) in the week following initial VT from 3-D US is reported along with AUC from ROC curve, and threshold is reported from ROC curve specificity/sensitivity maximum.

Measurement	Sensitivity (%)	Specificity (%)	AUC	Threshold used
VV	75	71.4	0.61	$<45.45\;{\mathrm{cm}}^{3}$
ΔVV	100	100	1	$<0.31\;{\mathrm{cm}}^{3}/\text{day}$

### 3.4 Comparison to 2-D US-Based Thresholds for Intervention

For patients who did not require interventions (Table 5), only 1 patient (IVH_P015) out of 20 was categorized incorrectly. Reported thresholds are based on the “bin” the test date fell in. In follow-up scans, this patient showed spontaneous resolution of PHVD. Interventional patients (Table 6) were all appropriately categorized using 3-D US VV thresholds. In general, they were enrolled in the study earlier and had earlier test dates to reflect that.

**Table 5:** Leave-one-out cross-validation for patients who did not have interventions based on clinical practice at our center. The calculated thresholds from leave-on-out cross-validation, the test day(s), whether the patient was above/below the threshold. Bolded values mean patient were miscatagorized.

Patient	Threshold	VV (${\mathrm{cm}}^{3}$)	Test age (days)
IVH_P002	<9.405	2.662	4
IVH_P004	<9.405	5.739	7
IVH_P005	<15.58	3.925	13
IVH_P006	<15.58	4.916	12
IVH_P009	<9.405	3.818	6
IVH_P014	<9.405	3.23	5
**IVH_P015**	**<10.57**	**15.38**	**9**
IVH_P016	<10.57	9.394	9
IVH_P018	<9.405	7.034	6
IVH_P020	<15.58	5.496	14
IVH_P021	<10.57	4.16	8
IVH_P042	<9.405	6.5	7
IVH_P044	<20.34	12	16
IVH_P046	<13.96	10.18	13
IVH_P047	<9.405	7.23	7
IVH_P049	<20.47	3.36	17
IVH_P050	<20.34	4.05	12
IVH_P054	<8.69	8.66	7
IVH_P058	<20.34	8.45	14
IVH_P059	<20.34	5.75	14

**Table 6:** Leave-one-out cross-validation for patients who had interventions based on clinical practice at our center. The calculated thresholds from leave-on-out cross-validation, the test day(s), whether the patient was above/below the threshold.

Patient	Threshold	VV (${\mathrm{cm}}^{3}$)	Test age (days)
IVH_P007	<10.57	26.14	9
IVH_P012	<9.35	10.15	4
IVH_P013	<9.405	15.15	7
IVH_P019	<10.57	16.66	8
IVH_P041	<9.405	10.21	5
IVH_P043	<10.57	15.907	8
IVH_P045	<9.405	20.4	7
IVH_P052	<10.57	17.41	8
IVH_P055	<9.405	44.75	7
IVH_P057	<10.78	11.43	8

Table 7 describes the age in days that each “interventional” neonate (N=14) in the study crossed the VI 97th centile +4  mm mark as well as when they crossed the 3-D US VV threshold reported in this study (described in Tables 6). On average, 3-D US VV could detect that an infant would receive an intervention 7.1 days before the infant reached the VI 97th centile +4  mm mark.

**Table 7:** The day of life infant crosses the threshold for potential intervention and the day of life the infant received intervention based on 2-D US VI 97th centile +4  mm+4 mm measurements and 3-D US VV measurements.

	VI 97th +4 mm	3-D US VV
Median (days)	15	7.1
Range (days)	2 to 29	4 to 9

## 4 Discussion

In our preliminary study, we presented the potential for 3-D US-based measurements of VVs from neonatal brain images to characterize whether or not patients with PHVD will receive interventions. Specifically, neonates who go on to receive interventions have larger VVs, {mean $21\pm 14\;{\mathrm{cm}}^{3}$ for patients who received interventions versus $5.6\pm 2.1\;{\mathrm{cm}}^{3}$ for patients who did not receive interventions during the first week of life [Fig. 1(a)]}. The patients who received interventions tend to have larger increases in VV change over time (Fig. 2).

In addition, we were able to show that our reported thresholds were able to predict that a neonate would receive an intervention 7 days before the use of the VI 97th centile +4  mm (Table 7). This could help give clinicians more time to inform parents of upcoming interventions as well as plan the interventions. This is particularly helpful if a neurosurgical consult needs to be brought into the clinical team to insert ventricular access devices (reservoirs) and/or ventriculosubgaleal shunts. In addition, if these results were to hold true in an independent sample of neonates with PHVD, it would grant an even earlier “early” time point should a study such as that performed in the ELVIS (early versus late ventricular interventional study) trial, which used the VI 97th centile +4  mm^24^ be performed using 3-D US VV.

3-D US-based VV acutely remains very high in the week following an intervention regardless of whether or not the patient’s PHVD is resolving (interventional patients had VV of $48.8\pm 12.5\;{\mathrm{cm}}^{3}$, whereas patients undergoing resolution had a VV of $45.1\pm 16.1\;{\mathrm{cm}}^{3}$). However, neonates who have PHVD that is resolving do show a change in VV ($-1.9\pm 1.3\;{\mathrm{cm}}^{3}/\text{day}$) from one 3-D imaging session to the next. This change is indicative of a reduction in VV or no change from previous image even in the week immediately after an intervention and is in comparison to the increase in VV in patients that receive additional VT ($2.4\pm 1.1\;{\mathrm{cm}}^{3}/\text{day}$). Though calculating the change in VV requires multiple imaging sessions, this may prove to be a better indicator than using VV alone for when to schedule therapies to drain CSF. IVH patients receiving interventions and certainly those receiving repeated VTs tend to have serial US images performed as part of clinical care. Furthermore, given the presented data, there is a high likelihood that even immediately after an initial VT even if the ventricles are stable or resolving, VV could very well be above a threshold VV. This could be due to how much CSF can be drained at any given VT. Removing larger amounts of CSF occasionally results in infants transiently appearing ashen, and in some cases experience a drop in blood pressure, heart rate, and oxygen saturation that is large enough requiring resuscitation after the procedure. To mediate adverse effects, knowledge of the VV increase following a tap might help better inform how much CSF to drain during subsequent interventions. Currently, it is not known how much CSF is optimal to drain and practices vary greatly among neurosurgeons; however, it is known that too little drained (<10  ml/kg of body weight) has no effect on ventricle size or ICP.^25^

Previous mathematical estimates of VV based on 2-D US images found that there was a significant difference between patients who received interventions and those who did not.^26^ This model received 2-D US images spaced at regular angular intervals and a calculation involving solving an integral using cylindrical coordinates.^27^ While fairly simplistic to solve for those with a background in calculus and geometry, this is a nontrivial level of knowledge to expect any given physician user to have. They found very good classification for patients who received treatment at 21 days of life using 30-mL VV or greater as a threshold, whereas we found a lower threshold of $20.9\;{\mathrm{cm}}^{3}$ at 21 to 27 days (Table 2). Brann et al.^26^ additionally found the change in VV was more predictive than VV alone with 100% sensitivity and specificity in both retrospective and prospective analysis, which concurs with the results of this study. Admittedly, change in VV is a more challenging metric as it requires multiple 3-D images. In addition, it should be noted that although this previous work was performed in the late 1980s, these criteria never made it into clinical practice. Perhaps the relative difficulty in generating this measurement was the reasons this method did not translate into general clinical practice. Perhaps there is less user error in the acquisition of a 3-D US image than attempting to acquire 2-D US images at equal angular spacing, and therefore, a 3-D US approach is more realistic to translate into practice.

Neonatal patients in the NICU are also at risk of having prenatal ventriculomegaly and this finding could be indicative of abnormalities such as neural tube defects or trisomy, which require follow-up after birth. For these neonates with larger than normal ventricles, the rate of change in VV might be a better indicator of which patients have progressive VD and not just large ventricles, which are stable in volume and are likely not to contribute to signs and symptoms of increases in ICP. Therefore, while our study did not include patients with prenatal ventriculomegaly, 3-D US VV should be examined in a cohort of patients with this indication, as it could impact how these patients’ care is managed.

The relative difficulty of generating VV through manual contouring a 3-D US image might prove too difficult for translation into clinical practice and this is a limitation of the current work. In light of this and to reduce the burden and length of time of manual ventricles segmentation from 3-D US images, we have developed both a semiautomated segmentation algorithm that can be used online at the bedside and an automated segmentation algorithm that can be run offline with no user input.^28^^,^^29^ Nevertheless, we used manual segmentation in the work reported here to minimize any errors due to algorithm segmentation, as change in VV would be very sensitive to these errors. Further evaluation of the algorithm segmentation methods with larger number of patients is required to determine best practices in the implementation of these new methods. The semiautomated segmentation algorithm has been integrated into our acquisition software and is being used in an ongoing pilot study to test and improve the method. We also speculate that a machine-learning approach may be superior to the methods we have developed and are willing to share our images and manual segmentations with investigators interested in developing such methods.

In segmenting the ventricles, the user did not use a purely boundary-based method as in some regions the boundaries can be difficult to detect or even not present. Since the user does not see a boundary all the time, they use prior knowledge of what shape the ventricle should be. As such, a combined approach that uses intensity information and searching for boundaries while including a shape prior has been our most successful algorithm.

Additional limitations of this work include the use of VT as the initial intervention and not using quantitative US measurements to determine interventional necessity. There is a wide variation among centers in whether the intervention is performed with lumbar puncture, VTs, ventricular reservoirs, third ventriculostomy, subgaleal shunts, and/or external ventricle drains. Further confounding the use of image-based metrics, 3-D US might be easier or harder to obtain depending on the ease of access to the fontanelle after different types of treatments have been performed. Furthermore, the criteria for intervention in this study are based on clinical judgment with multiple clinicians deciding on whether or not to tap, and not on quantitative measurements, which could hinder the reproducibility depending on the degree of clinical variation in practice. These limitations and questions of the validity of 3-D US-based VV in this patient population can only be elucidated in a larger study to account for greater variation in clinical practice.

As such, we have presented a potential clinical use for 3-D US within the NICU. This type of imaging appears to be promising to be able to help generate management strategies for neonates at risk of PHVD and the subsequent potential brain damage from elevated ICP. A larger, multicenter study is required to generate guidelines as this will increase the patient pool and better account for differences in clinical practice that occur in different centers.

## References

[1] Synnes A. R. et al., “Variations in intraventricular hemorrhage incidence rates among Canadian neonatal intensive care units,” J. Pediatr. 138, 525–531 (2001). BJNEEL 0268-8697 https://doi.org/10.1067/mpd.2001.111822 11295716 10.1067/mpd.2001.111822

[2] Papile L. A. et al., “Incidence and evolution of subependymal and intraventricular hemorrhage: a study of infants with birth weights less than 1500 gm,” J. Pediatr. 92, 529–534 (1978).https://doi.org/10.1016/S0022-3476(78)80282-0 305471 10.1016/s0022-3476(78)80282-0

[3] Christian E. A. et al., “Trends in hospitalization of preterm infants with intraventricular hemorrhage and hydrocephalus in the United States, 2000–2010,” J. Neurosurg. Pediatr. 17, 260–269 (2016).https://doi.org/10.3171/2015.7.PEDS15140 26544084 10.3171/2015.7.PEDS15140

[4] Zaben M. et al., “The initial neurosurgical interventions for the treatment of posthaemorrhagic hydrocephalus in preterm infants: a focused review,” Br. J. Neurosurg. 30, 7–10 (2016).https://doi.org/10.3109/02688697.2015.1096911 26468612 10.3109/02688697.2015.1096911

[5] Badhiwala J. H. et al., “Treatment of posthemorrhagic ventricular dilation in preterm infants: a systematic review and meta-analysis of outcomes and complications,” J. Neurosurg. Pediatr. 1–11 (2015).https://doi.org/10.3171/2015.3.PEDS14630 10.3171/2015.3.PEDS14630 26314206

[6] Whitelaw A. Aquilina K., “Management of posthaemorrhagic ventricular dilatation,” Arch. Dis. Child. Fetal Neonatal Ed. 97, F229–F223 (2012).https://doi.org/10.1136/adc.2010.190173 21289015 10.1136/adc.2010.190173

[7] Ingram M. C. et al., “Poor correlation between head circumference and cranial ultrasound findings in premature infants with intraventricular hemorrhage,” J. Neurosurg. Pediatr. 14, 184–189 (2014).https://doi.org/10.3171/2014.5.PEDS13602 24950469 10.3171/2014.5.PEDS13602

[8] Maunu J. et al., “Brain and ventricles in very low birth weight infants at term: a comparison among head circumference, ultrasound, and magnetic resonance imaging,” Pediatrics 123, 617–626 (2009). PEDIAU 0031-4005 https://doi.org/10.1542/peds.2007-3264 19171630 10.1542/peds.2007-3264

[9] Haiden N. et al., “3-D ultrasonographic imaging of the cerebral ventricular system in very low birth weight infants,” Ultrasound Med. Biol. 31, 7–14 (2005).https://doi.org/10.1016/j.ultrasmedbio.2004.07.017 15653225 10.1016/j.ultrasmedbio.2004.07.017

[r10] Gilmore J. H. et al., “Infant cerebral ventricle volume: a comparison of 3-D ultrasound and magnetic resonance imaging,” Ultrasound Med. Biol. 27, 1143–1146 (2001). USMBA3 0301-5629 https://doi.org/10.1016/S0301-5629(01)00400-8 11527602 10.1016/s0301-5629(01)00400-8

[r11] Riccabona M. et al., “Potential of three-dimensional ultrasound in neonatal and paediatric neurosonography,” Eur. Radiol. 13, 2082–2093 (2003).https://doi.org/10.1007/s00330-003-1845-4 12928958 10.1007/s00330-003-1845-4

[r12] Salerno C. C. et al., “Three-dimensional ultrasonographic imaging of the neonatal brain in high-risk neonates: preliminary study,” J. Ultrasound Med. 19, 549–555 (2000). JUMEDA 0278-4297 https://doi.org/10.7863/jum.2000.19.8.549 10944041 10.7863/jum.2000.19.8.549

[13] Abdul-Khaliq H. Lange P. E. Vogel M., “Feasibility of brain volumetric analysis and reconstruction of images by transfontanel three-dimensional ultrasound,” J. Neuroimaging 10, 147–150 (2000). JNERET 1051-2284 https://doi.org/10.1111/jon.2000.10.issue-3 10918740 10.1111/jon2000103147

[14] McLean G. et al., “Measurement of the lateral ventricles in the neonatal head: comparison of 2-D and 3-D techniques,” Ultrasound Med. Biol. 38, 2051–2057 (2012). USMBA3 0301-5629 https://doi.org/10.1016/j.ultrasmedbio.2012.07.018 23069135 10.1016/j.ultrasmedbio.2012.07.018

[r15] Romero J. M. et al., “Time efficiency and diagnostic agreement of 2-D versus 3-D ultrasound acquisition of the neonatal brain,” Ultrasound Med. Biol. 40, 1804–1809 (2014). USMBA3 0301-5629 https://doi.org/10.1016/j.ultrasmedbio.2014.03.013 24798394 10.1016/j.ultrasmedbio.2014.03.013

[16] Kim Y. J. et al., “Comparison between 3-dimensional cranial ultrasonography and conventional 2-dimensional cranial ultrasonography in neonates: impact on reinterpretation,” Ultrasonography (Seoul, Korea) 37 (1), 63–70 (2018).https://doi.org/10.14366/usg.17009 10.14366/usg.17009 PMC5769951 28780784

[17] Warf B. C., “Pediatric hydrocephalus in East Africa: prevalence, causes, treatments, and strategies for the future,” World Neurosurg. 73, 296–300 (2010).https://doi.org/10.1016/j.wneu.2010.02.009 20849782 10.1016/j.wneu.2010.02.009

[18] Kishimoto J. et al., “3D ultrasound system to investigate intraventricular hemorrhage in preterm neonates,” Phys. Med. Biol. 58, 7513–7526 (2013). PHMBA7 0031-9155 https://doi.org/10.1088/0031-9155/58/21/7513 24099882 10.1088/0031-9155/58/21/7513

[19] Kishimoto J. et al., “In vivo validation of a 3D ultrasound system for imaging the lateral ventricles of neonates,” Ultrasound Med. Biol. 42, 971–979 (2016). USMBA3 0301-5629 https://doi.org/10.1016/j.ultrasmedbio.2015.11.010 26782271 10.1016/j.ultrasmedbio.2015.11.010

[20] Fenster A. Downey D. B. Cardinal H. N., “Three-dimensional ultrasound imaging,” Phys. Med. Biol. 46, R67–R99 (2001). PHMBA7 0031-9155 https://doi.org/10.1088/0031-9155/46/5/201 11384074 10.1088/0031-9155/46/5/201

[21] Fenster A. Downey D., “3-D Ultrasound Imaging,” Ann. Rev. Biomed. Eng. 2, 457–475 (2000). ARBEF7 1523-9829 https://doi.org/10.1146/annurev.bioeng.2.1.457 11701520 10.1146/annurev.bioeng.2.1.457

[22] Brouwer M. J. et al., “New reference values for the neonatal cerebral ventricles,” Radiology 262, 224–233 (2012). RADLAX 0033-8419 https://doi.org/10.1148/radiol.11110334 22084208 10.1148/radiol.11110334

[23] Levene M. I., “Measurement of the growth of the lateral ventricles in preterm infants with real-time ultrasound,” Arch. Dis. Child. 56, 900–904 (1981). ADCHAK 0003-9888 https://doi.org/10.1136/adc.56.12.900 7332336 10.1136/adc.56.12.900 PMC1627506

[24] de Vries L. S. Brouwer A. J. Groenendaal F., “Posthaemorrhagic ventricular dilatation: when should we intervene?” Arch. Dis. Child. Fetal Neonatal Ed. 98, F284–F285 (2013).https://doi.org/10.1136/archdischild-2012-303158 23402888 10.1136/archdischild-2012-303158

[25] Kreusser K. L. et al., “Serial lumbar punctures for at least temporary amelioration of neonatal posthemorrhagic hydrocephalus,” Pediatrics 75, 719–724 (1985). PEDIAU 0031-4005 3885155

[26] Brann B. S. et al., “Measurement of progressive cerebral ventriculomegaly in infants after grades III and IV intraventricular hemorrhages,” J. Pediatr. 117, 615–621 (1990).https://doi.org/10.1016/S0022-3476(05)80701-2 2213391 10.1016/s0022-3476(05)80701-2

[27] Brann B. S. et al., “Quantification of neonatal cerebral ventricular volume by real-time ultrasonography. Derivation and in vitro confirmation of a mathematical model,” J. Ultrasound Med. 9, 1–8 (1990). JUMEDA 0278-4297 https://doi.org/10.7863/jum.1990.9.1.1 2404130 10.7863/jum.1990.9.1.1

[28] Qiu W. et al., “User-guided segmentation of preterm neonate ventricular system from 3-D ultrasound images using convex optimization,” Ultrasound Med. Biol. 41, 542–556 (2015). USMBA3 0301-5629 https://doi.org/10.1016/j.ultrasmedbio.2014.09.019 25542486 10.1016/j.ultrasmedbio.2014.09.019

[29] Qiu W. et al., “Automatic segmentation approach to extracting neonatal cerebral ventricles from 3D ultrasound images,” Med. Image Anal. 35, 181–191 (2016).https://doi.org/10.1016/j.media.2016.06.038 27428629 10.1016/j.media.2016.06.038

